# Pressure Drop Process as a Pretreatment for Enhancing Rehydration of Adzuki Beans (*Vigna angularis*)

**DOI:** 10.3390/foods14132286

**Published:** 2025-06-27

**Authors:** Suyeon Lee, Sangoh Kim, Seokwon Lim

**Affiliations:** 1 Department of Food Science & Biotechnology, Gachon University, 1342 Seongnam-daero, Sujeong-gu, Seongnam-si 13120, Gyeonggi-do, Republic of Korea; 2 Department of Food Engineering, Dankook University, 119, Dandae-ro, Dongnam-gu, Cheonan-si 31116, Chungcheongnam-do, Republic of Korea

**Keywords:** adzuki beans, pressure drop process, soaking, water absorption, microstructure

## Abstract

Pressure drop processes, such as dissolved inorganic carbon and gun-puffing, have shown utility in the food industry, but their reliance on heat remains a limiting factor. This study involved the development of a processor capable of performing nonthermal pressure drop treatment, which minimizes thermal changes in food. In addition, its effects on the structure and soaking efficiency of adzuki beans were analyzed. Two improved pressure drop processes were tested: PDA, which applied 1 kgf/cm^2^ of pressure before release, and PDB, which applied a higher pressure and gradually decreased it in steps of 1 kgf/cm^2^. Both the PDA and PDB pretreatments enhanced soaking more effectively than heat treatments at 60 °C and 100 °C, whereas no significant effect was observed at 25 °C, indicating a minimal heat requirement for moisture and gas release. Notably, repeated PDB application (more than 40 times) further increased the moisture absorption without thermal influence. Scanning electron microscopy revealed that the PDA, PDB, and heat treatments caused cracks in the hilum region and increased surface wrinkling and mesh structure deformation. These findings demonstrate the potential of pressure drop treatment to improve soaking efficiency through structural modification, supporting its use as an effective nonthermal pretreatment method.

## 1. Introduction

Adzuki beans (*Vigna angularis*) are legumes used in various foods, such as bean paste and porridge [1]. Adzuki beans have a hard surface and low moisture content, which are advantageous for storage. However, they have difficulty absorbing moisture, which is inconvenient during processing [2]. Therefore, grains and legumes, such as adzuki beans, are soaked before germination, fermentation, and cooking. Soaking is the process of immersing ingredients in water to absorb moisture, which uniformly denatures proteins and gelatinizes starch, resulting in a smooth texture after cooking [3]. If moisture is absorbed insufficiently, starch does not gelatinize properly, leaving a grainy texture after cooking [4]. Soaking requires a long time (approximately 12 h or more), and different types of grains and legumes require different soaking times, making it industrially inefficient [5]. To solve this problem, several methods have been investigated in the food industry to reduce soaking time.

Pressure drop processing techniques are effective in improving the physical and chemical properties of food [6,7,8]. In this technique the environment of a food product is rapidly reduced from high to low pressure, causing an explosive outflow of substances while changing its physical properties. It is performed in two steps. First, the sample is treated at high temperature and pressure, owing to which the gas inside the food is dissolves and the moisture expands. Next the pressure is rapidly reduced, the expanded moisture is released and the pores in the food expand, creating an environment where external substances are easily absorbed into the food. Based on this principle, pressure drop processes, such as instant controlled pressure drop (DIC) and gun-puffing, are currently used in the food industry for drying, texture enhancement, extraction, and other purposes. DIC involves short-term exposure to saturated vapor pressure at high temperatures (100–170 °C) followed by a sudden vacuum pressure drop to evaporate moisture, increase the diffusion of solvents, and release bioactive compounds from the food [9,10,11,12]. In gun-puffing, the pressure is increased above atmospheric pressure along with heating, and after attaining such pressure conditions, the pressure vessel is decompressed by opening its lid, which causes puffing of the food [13,14,15]. The conventional pressure drop process typically involves increasing the pressure by applying heat, which may lead to unintended side effects during pretreatment. Heat can inactivate proteins or gelatinize starch, resulting in alterations in the original characteristics of the food [16,17]. Additionally, accurately controlling the pressure is challenging, and consistently repeating the same process can be difficult. To overcome these disadvantages, a new pressure drop processor was designed and developed.

In the improved pressure drop process, low temperatures that do not deform the food are used, and the pressure drop is repeatedly applied to induce an impact on the surface of the adzuki beans. The anticipated advantages of this approach include precise control, the uniform treatment of all the samples, and the induction of cavitation. Precise control of the pressure allows the pressure drop process to be applied to food with delicate or fragile surfaces to achieve the desired effects. Moreover, because the pressure is applied uniformly to all the samples in the chamber, no specific area will be subjected to over- or under-treatment. In addition, repeated pressure drops can induce cavitation, which can have interesting effects. Cavitation is a phenomenon in which vapor cavities (bubbles) are formed within a fluid when the pressure falls below the vapor pressure, owing to rapid fluid movement. These bubbles are compressed and collapse as they move into higher pressure areas, generating strong impacts [18]. This process can produce noise and vibrations and even erode the surrounding objects. If part of the chamber briefly reaches a vacuum state during the pressure drop, cavitation may cause surface modifications of the adzuki beans.

Several nonthermal technologies, such as ultrasound and high-pressure processing (HPP), have been widely explored to enhance food quality and safety. However, ultrasound treatments often suffer from nonuniform effects owing to variations in the probe distance. Unlike HPP, which applies a single high-pressure treatment, the improved pressure drop process employs multiple treatments at relatively low pressures. Although these differences suggest that an improved pressure drop process may offer unique advantages, related studies are limited. Therefore, the objective of this study was to determine whether the improved pressure drop process can effectively modify the surface structure of adzuki beans, thereby enabling a more efficient soaking pretreatment. The soaking rate of and physical effects on adzuki beans treated at various temperatures, pressures, and repetitions of the pressure drop process were investigated. The results demonstrate the potential of the improved pressure drop process as a novel and innovative nonthermal technology for enhancing the efficiency of food processing operations.

## 2. Materials and Methods

### 2.1. Materials

Adzuki beans (*V. angularis*) were purchased from Hana Trading Co., Ltd. (Incheon, Republic of Korea) in 2023 and stored at 25 °C in an airtight environment.

### 2.2. Experimental Design

#### 2.2.1. Pressure Drop Processor

**Figure 1:**
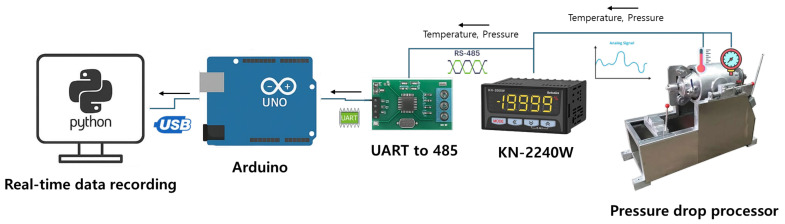
Pressure drop processor operation diagram.

The pressure drop processor was an electrically heated puffing machine (Mirae ENG, Yangju, Republic of Korea) equipped with temperature and pressure sensors. To store and analyze the temperature and pressure data in real time, a model KN-2240W (Autonics, Busan, Republic of Korea) was connected to an UNO R3 device (Arduino, Monza, Italy) via a universal asynchronous receiver/transmitter (UART) to an RS-485 communication module (Hiletgo, Shenzhen, China). A system was constructed in which data obtained via Modbus RTU communication at 9600 bp were transmitted to a PC through a USB connection. The sampling interval was set to 1 s. A Python program was created to record the temperature and pressure in real time using this setup (Figure 1).

**Figure 2:**
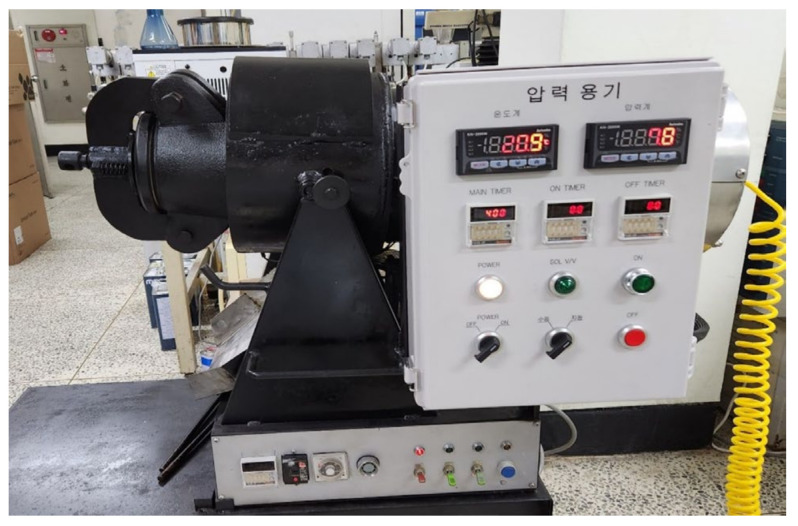
Pressure drop processor.

The pressure drop processor consists of an electric heater, pressure chamber, rotary motor, air compressor, and instrument panel. The maximum pressure that could be applied was 8 kgf/cm. The pressure chamber was made of cast iron (steel SS41-45C mixed) in the shape of a calabash, with an entrance for food and a body with an electric heater. The silicone lid at the entrance was lockable and releasable, allowing the pressure chamber to withstand internal pressures of up to 10 kgf/cm^2^. At the other end, a rubber hose connected to a locking valve allowed air to be injected from an air compressor to pressurize the chamber. The electric heater was regulated by a controller, and a rotary motor was built to rotate the pressure chamber to ensure an even heat distribution. The instrument panel was designed to display data from the temperature sensor and pressure sensor mounted inside the pressure chamber in units of °C and kgf/cm^2^, respectively, and a device was designed to automatically open and close the lock valve for a certain period (Figure 2).

When pressurized, the chamber can be released in two ways to allow the pressure to drop: through the locking valve and through a manually opened lid. The values measured by the sensor were recorded at 0.2 s intervals using a Python program with RS485 communication.

#### 2.2.2. Pressure Drop Process Methods

The pressure drop process using a pressure drop processor was performed on untreated adzuki beans. There are two types of pressure drop methods: pressure drop process A (PDA) and pressure drop process B (PDB). PDA involves pressurizing a pressure chamber to 1 kgf/cm^2^ (0.968 atm) and then forcing the lid to open and drop to atmospheric pressure. PDB involves pressurizing the pressure chamber to >1 kgf/cm^2^ and then releasing the pressure in steps of 1 kgf/cm^2^ through a locking valve, stopping at atmospheric pressure. In this study, after the pressure drop process, the samples were sealed and stored in conical tubes.

### 2.3. Observation of Hilum Damage

**Figure 3:**
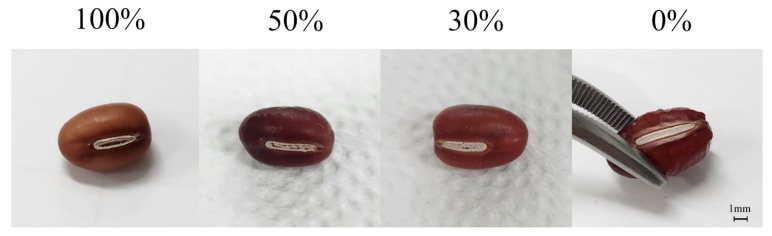
The extent of hilum damage in adzuki beans.

The degree of hilum damage was visually observed and classified as follows: 0% damage if there was no damage to the hilum; 30% damage if there were two or fewer holes; 50% damage if there were three or more holes; and 100% damage if more than half of the hilum was damaged (Figure 3). Holes were included only if they were 0.3 mm or larger.

### 2.4. Measurement of Moisture Content

Moisture content (water content) was determined at 160 °C with a model MB45 infrared moisture content meter (Ohaus Corporation, Parsippany, NJ, USA). In the soaking process, 30 g of adzuki beans were soaked in 150 mL of distilled water maintained at 25 °C for 12 h, and the progress was measured every 2 h from 0 h after soaking to 8 h. Approximately 3 g of the immersed adzuki beans was removed each time, the surface water was removed with a paper towel, and the measurement was performed.

### 2.5. Measurement of Water Activity

The water activity was measured at 25 °C with an AquaLab 4TE water activity meter (Meter Group Inc., Pullman, WA, USA). The soaking conditions were as follows: 30 g of adzuki beans were soaked in 150 mL of distilled water maintained at 25 °C for 12 h, and the progress was measured every 2 h from 0 h after soaking to 8 h. For each period, six soaked adzuki beans were removed and the surface water was removed with a paper towel. The beans were crushed for measurements [19].

### 2.6. Observation Using Scanning Electron Microscopy (SEM)

The surface of the sample was observed at ×100 and ×1000 magnifications by SEM using a model SU8600 device (Hitachi, Tokyo, Japan). The treated samples were dried in a desiccator at 60 °C for more than 48 h, crushed to extract the hilum portion, and then coated with platinum and palladium on carbon (PdPt) to make them conductive. The SEM images were analyzed using ImageJ software 1.54g (NIH, Bethesda, MD, USA) to measure the pore area of the hilum. The average pore radius was calculated as r = √(A/π).

### 2.7. Statistical Analyses

Statistical analyses were performed using SPSS Statistics (version 27.0, IBM, Armonk, NY, USA). One-way ANOVA variance was used to test for differences between group means, followed by Duncan’s multiple range test at a confidence level of *p* < 0.05. Statistically significant differences were indicated with red boxes in the graphs.

## 3. Results and Discussion

### 3.1. Pressure Drop Process

**Figure 4:**
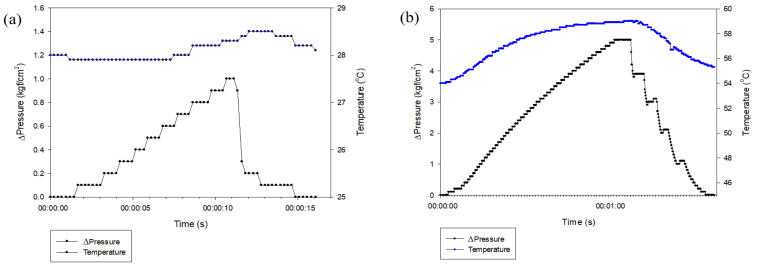
Temperature and pressure of (**a**) PDA and (**b**) PDB pressure drop process.

A record of the pressure drop over time is shown in Figure 4. The PDA is forced to drop the pressure through the entrance, so it decreases very rapidly at the beginning and decreases relatively slowly from 0.3 kgf/cm^2^ to reach atmospheric pressure. The temperature increases as pressure is applied and decreases as it drops, and the temperature difference during the drop is within 0.6–0.8 °C. The PDB used a locking valve to reduce the pressure. Therefore, the rate of pressure reduction was adjustable. When the pressure was high, the rate of decrease was rapid, and as the pressure difference decreased, the rate of decrease slowed. We also found that immediately after stopping the pressure drop, the decreasing pressure increased by 0.1–0.2 kgf/cm^2^. This suggests that the pressure inside the pressure chamber after a rapid pressure drop was not constant but fluctuated. Such internal pressure fluctuations may induce cavitation, particularly in the PDB process, where repeated pressure changes occur, unlike the single pressure drop in the PDA. In addition, because PDB starts from a higher initial pressure than PDA, it is presumed that greater structural modifications may occur during the treatment.

Although PDB showed a greater temperature change compared to PDA, with a difference of 4–5 °C at a maximum pressure of 5 kgf/cm^2^, its thermal stability was lower than that of PDA, which may be disadvantageous in thermal processes. However, when used in nonthermal applications, this difference is expected to have minimal impact.

### 3.2. Progression of Adzuki Beans over Soaking Time

**Figure 5:**
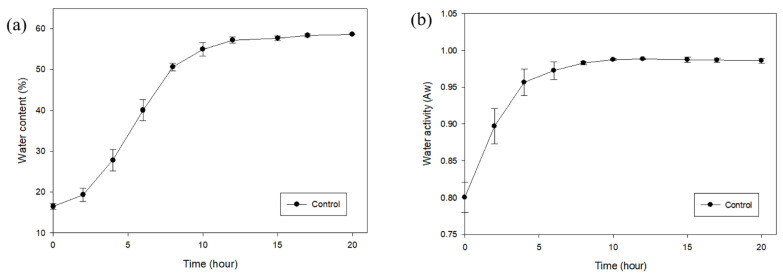
Moisture content (**a**) and water activity (**b**) of adzuki beans according to the soaking time.

The moisture content and water activity of the adzuki beans according to soaking time are shown in Figure 5. The water content increased over time in the form of a sigmoidal graph, with no significant increase from 0 to 4 h, a rapid increase from 4 to 10 h, and a decrease in speed after 10 h to reach water absorption equilibrium. The findings were similar to the results of a previous study [20]. The water activity increased steadily from 0 to 6 h, and then remained constant after 6 h, indicating that water absorption equilibrium had been reached.

**Figure 6:**
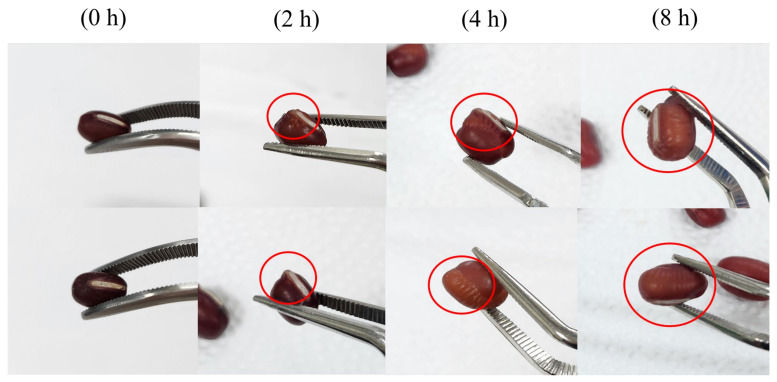
Changes in the appearance of adzuki beans according to the soaking time.

Figure 6 shows the changes in the appearance of the adzuki beans according to the soaking time. After 0 h, no water absorption was apparent, whereas after 2 h, the hilum expanded due to water absorption from the edges, as indicated by the areas marked with red circles. After 4 h of soaking, more wrinkles were observed around the hilum, along with further swelling of the surface, and a lighter color of the epithelium. At 6 h of soaking, almost all of the area had absorbed moisture, and overall wrinkling and lightening of the color could be seen. These findings suggest that water absorption begins at the periphery of the hilum, consistent with the results of legume soaking studies [21,22].

### 3.3. Hilum Damage in Hydrated Adzuki Beans

The adzuki beans with high moisture absorption were evenly expanded, whereas those with low moisture absorption were wrinkled and not fully hydrated. The percentages of damage to the adzuki beans with high and low moisture absorption are shown in Table 1. The highly moisturized adzuki beans had 50% damage in 50.0% of the beans and 100% damage in 27.8% of the total, with more than half of the beans having 50% and 100% damage, respectively. The adzuki beans with low moisture absorption accounted for 5.9% each of the 50% and 100% damaged beans, with more than 50% having ≤30% damage. It can be assumed that the greater the damage to the hilum, the faster the moisture absorption, because moisture is absorbed from around the hilum. One limitation of this study is that the hilum damage was assessed solely through visual inspection. There was no quantitative measurement. Therefore, a statistical correlation or regression analysis between the degree of hilum damage and water absorption or activity could not be performed. Future studies incorporating image analysis or mechanical assessment to quantify hilum integrity may allow for more robust statistical modeling.

### 3.4. Hilum Damage in PDA-Treated Adzuki Beans

**Figure 7:**
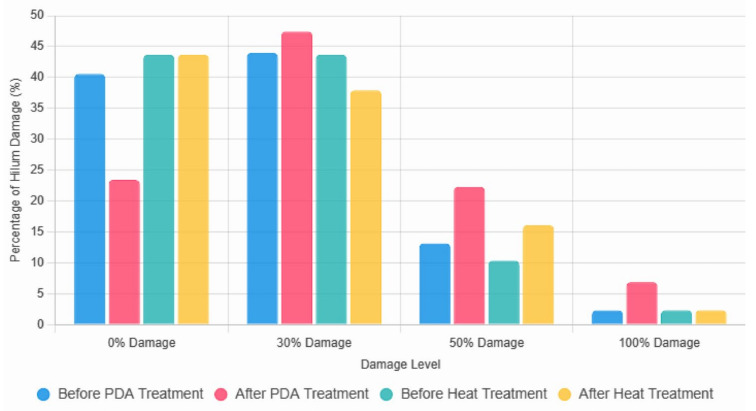
Damage to the hilum of adzuki beans before and after PDA and heat treatment.

The damage to the adzuki bean hilum before and after the PDA treatment and heat treatment at the same temperature and in the same time conditions are shown in Table 2 and Figure 7. The heat treatment condition was 100 °C for 10 min, and the PDA treatment condition was 100 °C for 10 min, repeated 10 times. Before the PDA treatment, a 0% damage level was seen in 40.57% of the total. After the PDA treatment, it was 23.43%, representing a total reduction of 17.14%. The proportion of beans with 0% damage increased by 3.43% (from 44.00% to 47.43%), whereas those with 50% damage increased by 9.14% (from 13.14% to 22.29%), and those with 100% damage increased by 4.57% (from 2.29% to 6.86%). The heat-treated adzuki beans remained unchanged, from 0% damage before treatment to 43.68%. The proportion of beans with 30% damage decreased by 5.75% (from 43.68% to 37.93%), and those with 50% damage increased by 5.75% from 10.34% to 16.09%, while the 100% damage rate remains unchanged at 2.30%. The results show that both the heat and PDA treatments were effective in increasing the damage level of the hilum. In particular, the heat treatment increased the damage level, whereas the PDA treatment had a greater effect. It can be concluded that PDA treatment at 100 °C is more effective in increasing hilum damage. However, a high level of damage does not necessarily guarantee improved water absorption. This suggests that when the hilum damage exceeds a certain threshold, excessive disruption of the starch granules or protein matrix in the adzuki bean may reduce water absorption efficiency [3,5]. Therefore, it is presumed that in the case of 100% hilum damage, where more than half of the hilum structure is damaged, the overall structure of the beans may be excessively compromised.

### 3.5. Moisture Content and Water Activity of PDA-Treated Adzuki Beans Under Different Conditions

**Figure 8:**
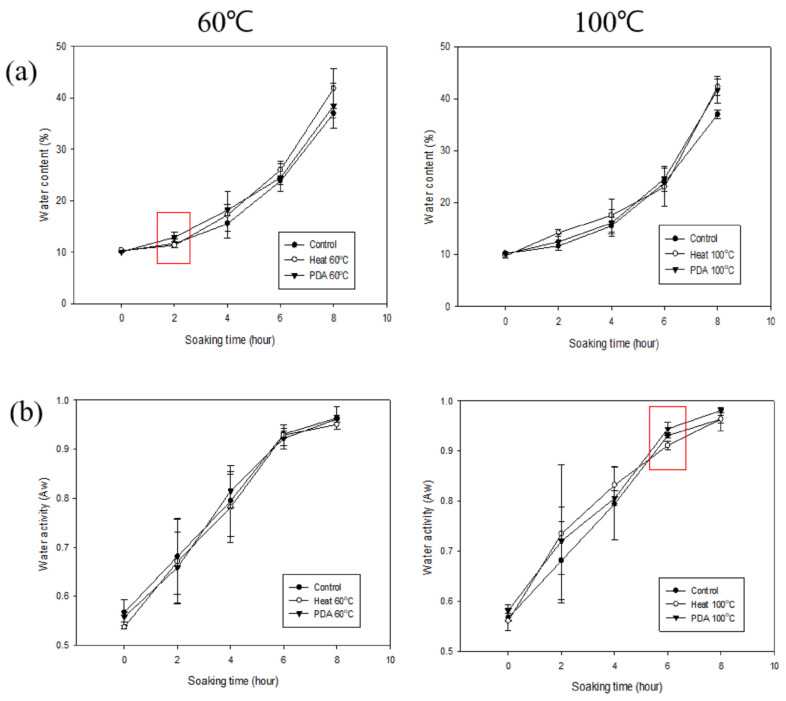
The water content (**a**) and water activity (**b**) of untreated and heat-treated (60 and 100 °C) and PDA-treated (25, 60, and 100 °C) adzuki beans.

Since the hilum damage to the PDA-treated adzuki beans at 100 °C increased, we performed an experiment to determine whether soaking was effective. The PDA treatment conditions were repeated 20 times at 25, 60, and 100 °C for 20 min each. For comparison, the heat treatment was performed at 60 and 100 °C for 20 min each. The water content and activity of adzuki beans soaked for 0–8 h after pretreatment were investigated (Figure 8). The moisture content of the control group was 10.21 ± 0.27%, 11.58 ± 0.80%, 15.55 ± 1.56%, 24.12 ± 0.82%, and 37.20 ± 0.94% at 0, 2, 4, 6, and 8 h of soaking, respectively (Table 3). In the 100 °C heat-treated group, the moisture content was 14.19 ± 3.17% at 2 h, which was significantly higher than that of the control. In the 60 °C PDA-treated group, the moisture content was 12.91 ± 0.91% at 2 h, which was significantly higher than that of the 60 °C heat-treated group. The water activity of the control group increased gradually over time, reaching 0.9637 ± 0.0233 at 8 h (Table 4). In the 100 °C PDA-treated group, the water activity at 6 h (0.9443 ± 0.0131) was significantly higher than that of the 100 °C heat-treated group at the same time point. Although most of the results were not significantly different from those of the control group, the PDA-treated adzuki beans exhibited a slightly faster soaking rate than the heat-treated beans at the same temperature. However, PDA treatment at 100 °C did not lead to a clear improvement in hydration, despite increasing hilum damage. This aligns with our earlier observation that excessive hilum damage may reduce water absorption efficiency. Therefore, the structural alterations caused by the high-intensity PDA treatment may have compromised the internal matrix of the adzuki bean, hindering, rather than enhancing, water uptake.

**Figure 9:**
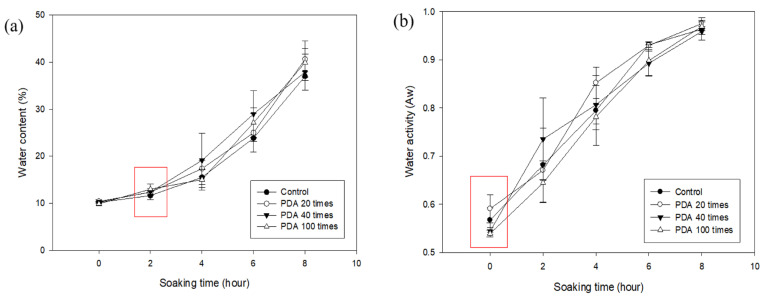
The water content (**a**) and water activity (**b**) of untreated and PDA-treated (20, 40, and 100 times) adzuki beans.

The moisture content and water activity of the adzuki beans treated with PDA (25 °C, 20, 40, 100 times) and the control group are shown in Figure 9, Table 5 and Table 6. The beans treated with PDA 100 times had a moisture content of 13.02 ± 1.08% at 2 h soaking, which was significantly higher than the control. The water activity for this PDA treatment ranged from 0.5390 ± 0.0071 (0 h) to 0.9693 ± 0.0092 (8 h) and was not significantly different from the control, but it was significantly lower than the value following the treatment conducted 20 times. The PDA 100-time treatment enhanced the initial moisture absorption at 2 h but showed no sustained effect over longer soaking times (4–8 h), suggesting that structural changes caused by the PDA may limit prolonged moisture uptake. The lower water activity in the PDA 100-time treatment compared to the 20-time treatment indicates increased moisture evaporation due to the repeated pressure drops, which enhances drying. However, it did not significantly affect the soaking efficiency compared to the control. The PDA 100-time treatment slightly improved the early moisture absorption, but excessive treatments (more than 40 times) increased the drying without enhancing the long-term soaking efficiency. Optimizing the PDA for 40 or fewer cycles may be more effective.

**Table 6:** Water activity of untreated and PDA-treated (20, 40, and 100 times) adzuki beans.

Soaking Time (h)	Water Activity (Aw)			
	Control	PDA 20 Times	PDA 40 Times	PDA 100 Times
0	0.5671 ± 0.0258 ^ab^	0.5906 ± 0.0294 ^b^	0.5438 ± 0.0079 ^a^	0.5390 ± 0.0071 ^a^
2	0.6812 ± 0.0773 ^a^	0.6704 ± 0.0197 ^a^	0.7352 ± 0.0858 ^a^	0.6442 ± 0.0407 ^a^
4	0.7945 ± 0.0727 ^a^	0.8522 ± 0.0329 ^a^	0.8067 ± 0.0403 ^a^	0.7815 ± 0.0268 ^a^
6	0.9310 ± 0.0054 ^a^	0.9298 ± 0.0083 ^a^	0.8927 ± 0.0249 ^a^	0.8984 ± 0.0324 ^a^
8	0.9637 ± 0.0233 ^a^	0.9752 ± 0.0062 ^a^	0.9588 ± 0.0055 ^a^	0.9693 ± 0.0092 ^a^

All values are mean ± SD. ^a,b^ Values in the same row with different superscript letters are significantly different (*p* < 0.05).

### 3.6. Moisture Content and Water Activity of PDB-Treated Adzuki Beans Under Different Conditions

**Figure 10:**
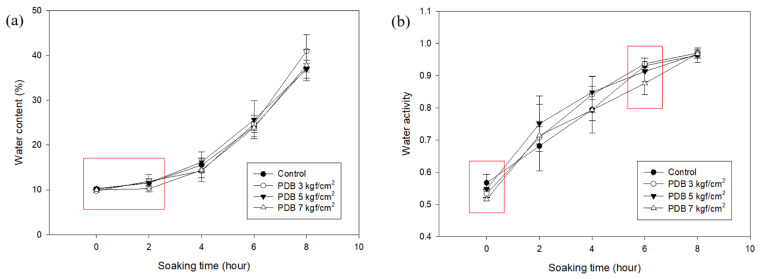
The water content (**a**) and water activity (**b**) of untreated and PDB-treated (3, 5, and 7 kgf/cm^2^) adzuki beans.

The results of the experiment that was performed to investigate the difference in the moisture content of PDB-treated adzuki beans at maximum pressures of 3, 5, and 7 kgf/cm^2^ are shown in Figure 10, Table 7 and Table 8.

The maximum pressure of the PDB, which was pressurized to the maximum pressure and then lowered by 1 kgf/cm^2^ to stop at atmospheric pressure, was 3, 5 and 7 kgf/cm^2^. The moisture contents of the adzuki beans treated with PDB at 3 kgf/cm^2^ were 9.77 ± 0.27% (0 h), 11.96 ± 1.59% (2 h), 14.23 ± 1.53% (4 h), 24.73 ± 1.91% (6 h), and 40.96 ± 3.61% (8 h). All the values were significantly lower than the control at 0 h. The respective values at 0, 2, 4, 6, and 8 h for the beans treated with PDB 7 kgf/cm^2^ were 10.00 ± 0.07%, 10.26 ± 0.68%, 14.41 ± 2.55%, 23.94 ± 2.07%, and 37.80 ± 3.48%. These values were similar to the control values but significantly lower than PDB 3 kgf/cm^2^ at 2 h.

The water activity values for the adzuki beans treated with PDB 3 kgf/cm^2^ at 0, 2, 4, 6, and 8 h were 0.5335 ± 0.0122, 0.7076 ± 0.1036, 0.8414 ± 0.0560, 0.9371 ± 0.0175, and 0.9704 ± 0.0042. These values were significantly lower than the control at 0 h. For the beans treated with PDB 7 kgf/cm^2^, the water activity values at the same respective times were 0.5154 ± 0.0048, 0.7144 ± 0.0264, 0.7933 ± 0.0330, 0.8762 ± 0.0356, and 0.9682 ± 0.0144. These values were significantly lower than the control at 0 and 6 h. Both the moisture content and water activity values were generally lower than the control in the beans treated with PDB 7 kgf/cm^2^. The findings suggest the absence of a soaking effect. Treatment with PDB 3 kgf/cm^2^ was lower than that of the control at 0 h of soaking because it was not possible to form a structure that could better absorb moisture. Therefore, only moisture near the surface was allowed to evaporate. The lack of enhanced moisture absorption in the adzuki beans treated with 7 kgf/cm^2^ PDB may be attributed to excessive structural damage or compaction of the seed coat and hilum. The higher pressure could have caused overcompression or collapse of the porous network within the bean, limiting water penetration. Based on these results, 5 kgf/cm^2^ is considered an appropriate level for PDB implementation, and treatment with PDB 10 times at 25 °C did not have a significant effect on soaking.

**Figure 11:**
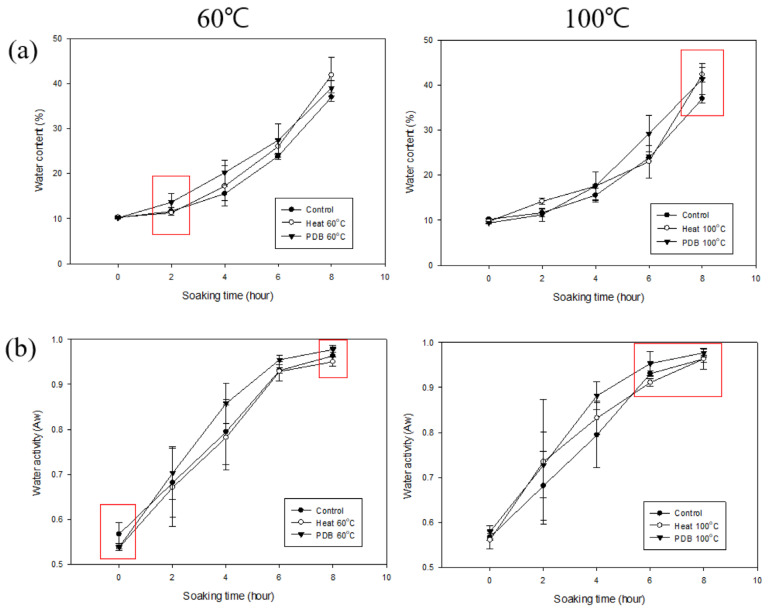
The water content (**a**) and water activity (**b**) of untreated and heat-treated (60 and 100 °C) and PDB-treated (25, 60, and 100 °C) adzuki beans.

Since PDB at 25 °C was ineffective for soaking, experiments were performed to determine the effect of PDB treatment at different temperatures. The PDB treatment conditions were 25, 60, and 100 °C for 20 min each, with 10 repetitions. Heat treatment at 60 and 100 °C for 20 min was carried out for comparison (Figure 11).

The moisture content of the adzuki beans heat-treated at 60 °C was 10.33 ± 0.26% (0 h), 11.31 ± 0.08% (2 h), 17.25 ± 4.50% (4 h), 26.00 ± 1.64% (6 h), and 41.84 ± 3.94% (8 h). All the values were significantly higher than the control at 8 h (Table 9). The values for the 100 °C heat treatment were 9.75 ± 0.43% (0 h), 14.19 ± 3.17% (2 h), 17.58 ± 3.17% (4 h), 23.01 ± 3.61% (6 h), and 42.31 ± 1.59% (8 h). These values were significantly higher than the control at 2 and 8 h. The values for the PDB treatment at 60 °C were 10.18 ± 0.16% (0 h), 13.64 ± 1.89% (2 h), 20.22 ± 2.75% (4 h), 27.40 ± 3.65% (6 h), and 38.95 ± 1.71% (8 h). These values were significantly different from the control at 2 h. Finally, the values for the PDB treatment at 100 °C were 9.40 ± 0.26% (0 h), 11.24 ± 1.50% (2 h), 17.58 ± 3.07% (4 h), 29.21 ± 4.04% (6 h), and 41.33 ± 3.43% (8 h). These values were significantly lower than the control at 0 h, higher at 8 h, and higher than the 100 °C heat-treated beans at 6 h.

The water activity of the adzuki beans heat-treated at 60 °C was 0.5374 ± 0.0040 (0 h), 0.6709 ± 0.0865 (2 h), 0.7821 ± 0.0720 (4 h), 0.9288 ± 0.0213 (6 h), and 0.9502 ± 0.0100 (8 h). All the values were significantly lower than the control at 0 h (Table 10). The values following the heat treatment with PDB at 60 °C were 0.5385 ± 0.0074 (0 h), 0.7027 ± 0.0586 (2 h), 0.8578 ± 0.0452 (4 h), 0.9548 ± 0.0106 (6 h), and 0.9775 ± 0.0058 (8 h). The values were lower than the control at 0 h but higher at 8 h, and higher than those of the 60 °C heat treatment. The values following the treatment with PDB at 100 °C were 0.5792 ± 0.0043 (0 h), 0.7275 ± 0.0730 (2 h), 0.8819 ± 0.0309 (4 h), 0.9534 ± 0.0270 (6 h), and 0.9773 ± 0.0085 (8 h). These values were significantly higher than the control at 8 h and higher than 100 °C heat treatment at 6 h.

Based on the moisture content and water activity findings, PDB treatment appears more effective for soaking than treatments at 60 and 100 °C.

**Figure 12:**
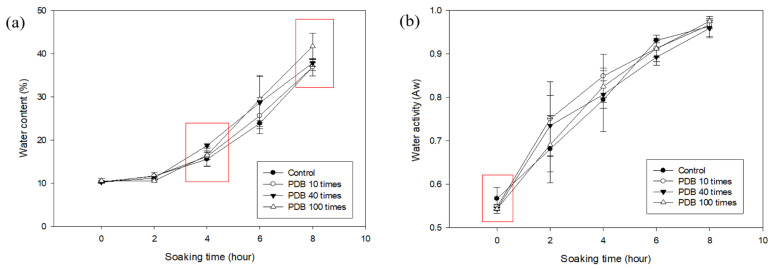
The water content (**a**) and water activity (**b**) of untreated and PDB-treated (10, 40, and 100 times) adzuki beans.

The moisture content and water activity for the PDB treatment at 25 °C with 10, 40, and 100 repetitions are shown in Figure 12, Table 11 and Table 12.

The moisture content values for 40 cycles were 10.22 ± 0.23% (0 h), 11.17 ± 0.20% (2 h), 18.71 ± 0.60% (4 h), 28.66 ± 6.07% (6 h), and 37.90 ± 0.77% (8 h). All the values were higher than the control at 4 h. The values for 100 cycles were 10.54 ± 0.46% (0 h), 10.53 ± 0.24% (2 h), 16.48 ± 0.98% (4 h), 29.47 ± 5.49% (6 h), and 41.76 ± 3.04% (8 h). The values were significantly different from the control at 8 h. The water activity values for 40 cycles were 0.5355 ± 0.0043 (0 h), 0.6988 ± 0.0693 (2 h), 0.8317 ± 0.0316 (4 h), 0.8904 ± 0.0187 (6 h), and 0.9716 ± 0.0223 (8 h). All the values were lower than the control at 0 h. Correlation analysis between the number of cycles and moisture content at 8 h showed a positive relationship, indicating that increased PDB cycles generally enhanced moisture uptake. However, the difference in moisture gain between 40 and 100 cycles was relatively small, suggesting a potential saturation point in the efficacy of the repeated pressure drop treatment. This may be due to the limited capacity of the seed structure to accommodate additional water, or a plateau in the disruption of the seed surface. Moreover, it is possible that excessive PDB cycles compromise structural integrity or promote the leaching of internal solutes, which could reduce the net effectiveness of soaking enhancement. Further microstructural or compositional analyses would be helpful in confirming these effects. A similar trend was observed in the PDA-treated samples, where the soaking performance did not markedly improve beyond 40 cycles, suggesting consistent results across both treatment types. These findings indicate that although repeated PDB and PDA treatments can enhance soaking performance, more than 40 cycles may yield diminishing returns.

### 3.7. SEM Surface Analysis of Adzuki Beans Pretreated Using the Pressure Drop Process

**Figure 13:**
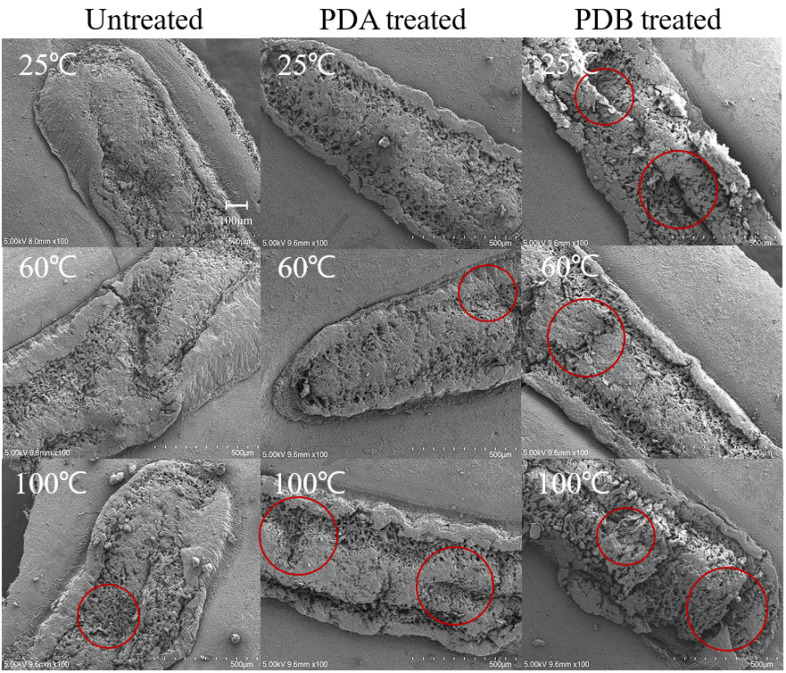
Hilum microstructure of adzuki beans treated with heat, PDA, and PDB; 100× magnification.

**Figure 14:**
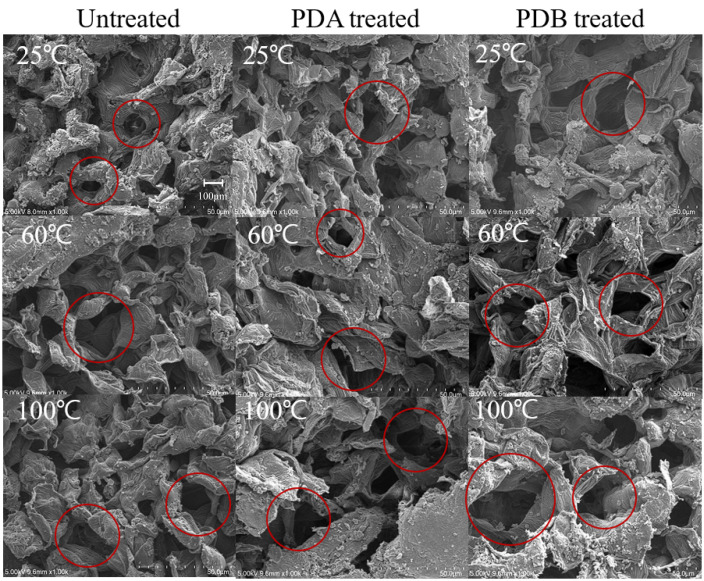
Hilum microstructure of adzuki beans treated with heat, PDA, and PDB; 1000× magnification.

**Figure 15:**
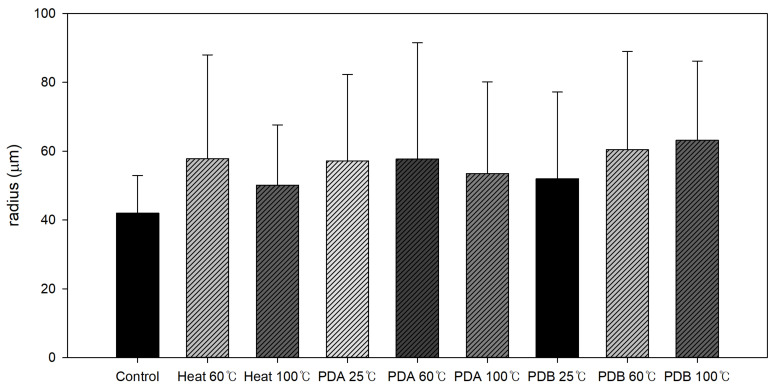
Structural changes in hilum pore radius with PDA, PDB, and heat treatments.

SEM was used to examine the surface differences among control and heat-, PDA-, and PDB-treated adzuki beans. At 100× magnification, the control hilum showed a membrane-like substance in some areas and a net-like mesh structure in uncovered areas (Figure 13). The heat-, PDA-, and PDB-treated hila were cracked with increased mesh structure. Notably, the hilum treated with PDA at 100 °C and PDB at 25, 60, and 100 °C appeared swollen or collapsed, likely due to the escape of water or volatiles during the pressure drop process, as indicated by the areas marked with red circles. At 1000× magnification, the control sample’s mesh structure had pores of 80–100 μm, while treated samples (heat, PDA, and PDB) showed larger pores (100–200 μm), indicating that these treatments not only damaged the hilum but also enlarged the mesh structure (The area marked by red circles in Figure 14 and Figure 15). This increase has been reported to positively affect hydration, as supported by structural change studies using DIC [23,24,25,26]. The SEM images confirmed that PDA, PDB, and DIC led to an enlarged mesh structure and increased pore sizes (100–200 μm in this study vs. 100–300 μm in a previous study [23]). These microstructural changes are known to enhance water uptake by increasing the surface area and forming capillary-like channels that facilitate faster water diffusion into core tissues, as reported for DIC-treated plant materials. Consistent with this, the moisture content after soaking (e.g., 41.76% at 8 h for PDB 100 times) aligns with the 30–50% rehydration range reported elsewhere [23,25], and the water activity values (e.g., 0.9716 for PDB 40 times) fall within the 0.9–0.98 range previously reported [26]. These findings indicate effective hydration and water retention. However, hydration limitations have also been observed. Despite the structural expansion, the adzuki beans showed a slower initial moisture uptake than DIC-treated fruits or mushrooms, likely because of their dense, starch-rich cellular matrix. Additionally, while DIC at higher temperatures (80–120 °C) significantly improved rehydration in previous studies, PDB at 25 °C was less effective, underscoring the role of thermal input alongside structural changes in enhancing water absorption.

**Figure 16:**
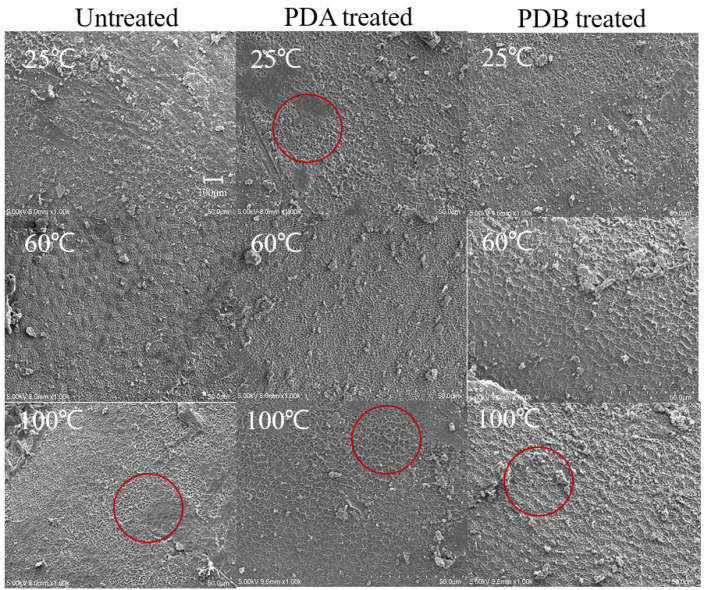
Surface microstructure of adzuki beans treated with heat, PDA, and PDB; 1000× magnification.

Representative views of the surfaces of the adzuki beans evident at 1000× magnification are shown in Figure 16. When observing the control group, we noticed wrinkles on the surface around the hilum. This phenomenon was also observed when other samples were observed over a larger area than the untreated samples, as indicated by the red circles. It was assumed that the wrinkles were caused by damage to the adzuki bean surface layer. Because the wrinkles increased after the treatment, it can be presumed that some force was applied to the surface layer, causing some of the surface to peel off and reveal wrinkles.

## 4. Conclusions

In this study, the effects of improved pressure drop treatments on the structural changes and soaking behavior of adzuki beans were investigated. The moisture content and water activity values after soaking showed that PDA pretreatment at 60 and 100 °C had no significant advantage over thermal treatment alone. In contrast, PDB pretreatment under the same conditions resulted in a greater enhancement in soaking compared to both thermal treatment and the control. These findings suggest that minimal thermal input during the pressure drop process is necessary to facilitate the release of internal moisture and volatile gases, contributing to the formation of pores that improve soaking efficiency. At 25 °C, PDA did not produce significant soaking improvement, even with repeated treatment. In contrast, PDB treatment performed 40 times or more resulted in a measurable increase in moisture content. These findings indicate that the PDB method can enhance soaking efficiency through pressure variation alone, without the need for additional heat. However, when the treatment was repeated 100 times, signs of structural damage were observed, suggesting that 40 PDB cycles may be the most appropriate. SEM observations of the hilum region of the adzuki beans following the heat, PDA, and PDB pretreatments revealed increased crack formation and the enlargement of mesh-like pores. Specifically, beans treated with PDA and PDB at 100 °C displayed swollen or collapsed mesh structures. In addition, the surfaces of beans treated with PDA and PDB showed more pronounced wrinkling than the surface of the control beans. These results indicate that the pressure drop process in a heated environment affects the hilum structure and induces physical stress on the surface. Future studies should aim to identify the optimal number of pressure drop cycles to maximize soaking efficiency without inducing structural damage, as the results of this study suggest diminishing returns beyond 40 cycles. Additionally, combining pressure drop methods, particularly the PDB approach, with other nonthermal technologies, such as ultrasound or pulsed electric fields, could further enhance water absorption by creating synergistic effects on the cellular structure. Investigating such hybrid approaches, along with real-time microstructural analysis and nutrient retention evaluation, would offer valuable insights into the development of efficient and scalable soaking pretreatments for legumes and grains.

## Figures and Tables

**Table 1:** Damage to hilum of hydrated adzuki beans.

Damage to Hilum (%)	Completely Hydrated Adzuki Beans (%)	Incompletely Hydrated Adzuki Beans (%)
0	0.0	47.1
30	22.2	41.2
50	50.0	5.9
100	27.8	5.9

Values indicate proportions based on visual inspection (sample size, n = 18).

**Table 2:** Damage to hilum of adzuki beans before and after PDA and heat treatment.

Damage to Hilum (%)	Before PDA Treatment (%)	After PDA Treatment (%)	Before Heat Treatment (%)	After Heat Treatment (%)
0	40.57	23.43	43.68	43.68
30	44.00	47.43	43.68	37.93
50	13.14	22.29	10.34	16.09
100	2.29	6.86	2.30	2.30

Values indicate proportions based on visual inspection (sample size, n = 175).

**Table 3:** Moisture content of untreated and heat-treated (60 and 100 °C) and PDA-treated (25, 60, and 100 °C) adzuki beans.

Soaking Time	Moisture Content (%)					
	Control	Heat 60 °C	Heat 100 °C	PDA 25 °C	PDA 60 °C	PDA 100 °C
0 h	10.21 ± 0.27 ^ab^	10.33 ± 0.26 ^b^	9.75 ± 0.43 ^a^	10.42 ± 0.32 ^b^	10.05 ± 0.20 ^ab^	10.15 ± 0.08 ^ab^
2 h	11.58 ± 0.80 ^ab^	11.31 ± 0.08 ^a^	14.19 ± 3.17 ^c^	12.36 ± 0.38 ^ab^	12.91 ± 0.91 ^bc^	12.47 ± 1.60 ^ab^
4 h	15.55 ± 1.56 ^a^	17.25 ± 4.50 ^a^	17.58 ± 3.17 ^a^	17.39 ± 1.67 ^a^	18.20 ± 1.05 ^a^	16.09 ± 2.64 ^a^
6 h	24.12 ± 0.82 ^a^	26.00 ± 1.64 ^a^	23.01 ± 3.61 ^a^	25.00 ± 4.05 ^a^	24.51 ± 2.69 ^a^	24.64 ± 2.41 ^a^
8 h	37.20 ± 0.94 ^a^	41.84 ± 3.94 ^a^	42.31 ± 1.59 ^a^	40.62 ± 3.92 ^a^	38.46 ± 4.41 ^a^	41.73 ± 2.63 ^a^

All values are mean ± SD. ^a,b,c^ Values in the same row with different superscript letters are significantly different (*p* < 0.05).

**Table 4:** Water activity of untreated and heat-treated (60 and 100 °C) and PDA-treated (25, 60, and 100 °C) adzuki beans.

Soaking Time	Water Activity (Aw)					
	Control	Heat 60 °C	Heat 100 °C	PDA 25 °C	PDA 60 °C	PDA 100 °C
0 h	0.5671 ± 0.0258 ^ab^	0.5374 ± 0.0040 ^a^	0.5612 ± 0.0015 ^ab^	0.5906 ± 0.0294 ^b^	0.5584 ± 0.0099 ^ab^	0.5816 ± 0.0054 ^b^
2 h	0.6812 ± 0.0773 ^a^	0.6709 ± 0.0865 ^a^	0.7350 ± 0.1382 ^a^	0.6704 ± 0.0197 ^a^	0.6589 ± 0.0718 ^a^	0.7207 ± 0.0671 ^a^
4 h	0.7945 ± 0.0727 ^a^	0.7821 ± 0.0720 ^a^	0.8323 ± 0.0372 ^a^	0.8522 ± 0.0329 ^a^	0.8150 ± 0.0341 ^a^	0.8059 ± 0.0143 ^a^
6 h	0.9310 ± 0.0054 ^ab^	0.9288 ± 0.0213 ^ab^	0.9112 ± 0.0083 ^a^	0.9298 ± 0.0083 ^ab^	0.9220 ± 0.0212 ^ab^	0.9443 ± 0.0131 ^b^
8 h	0.9637 ± 0.0233 ^ab^	0.9502 ± 0.0100 ^a^	0.9637 ± 0.0080 ^ab^	0.9752 ± 0.0062 ^b^	0.9613 ± 0.0072 ^ab^	0.9810 ± 0.0041 ^b^

All values are mean ± SD. ^a,b^ Values in the same row with different superscript letters are significantly different (*p* < 0.05).

**Table 5:** Moisture content of untreated and PDA-treated (20, 40, and 100 times) adzuki beans.

Soaking Time, h	Moisture Content (%)			
	Control	PDA 20 Times	PDA 40 Times	PDA 100 Times
0	10.21 ± 0.27 ^a^	10.42 ± 0.32 ^a^	10.32 ± 0.14 ^a^	9.91 ± 0.46 ^a^
2	11.58 ± 0.80 ^a^	12.36 ± 0.38 ^ab^	12.41 ± 0.76 ^ab^	13.02 ± 1.08 ^b^
4	15.55 ± 1.56 ^a^	17.39 ± 1.67 ^a^	19.15 ± 5.75 ^a^	15.05 ± 2.22 ^a^
6	24.12 ± 0.82 ^a^	25.00 ± 4.05 ^a^	28.99 ± 4.98 ^a^	27.14 ± 3.17 ^a^
8	37.20 ± 0.94 ^a^	40.62 ± 3.92 ^a^	37.88 ± 3.80 ^a^	39.89 ± 2.97 ^a^

All values are mean ± SD. ^a,b^ Values in the same row with different superscript letters are significantly different (*p* < 0.05).

**Table 7:** Moisture content of untreated and PDB-treated (3, 5, and 7 kgf/cm^2^) adzuki beans.

Soaking Time, h	Moisture Content (%)			
	Control	PDB 3 kgf/cm^2^	PDB 5 kgf/cm^2^	PDB 7 kgf/cm^2^
0	10.21 ± 0.27 ^b^	9.77 ± 0.27 ^a^	10.29 ± 0.13 ^b^	10.00 ± 0.07 ^ab^
2	11.58 ± 0.80 ^ab^	11.96 ± 1.59 ^b^	11.61 ± 0.87 ^ab^	10.26 ± 0.68 ^a^
4	15.55 ± 1.56 ^a^	14.23 ± 1.53 ^a^	16.19 ± 2.26 ^a^	14.41 ± 2.55 ^a^
6	24.12 ± 0.82 ^a^	24.73 ± 1.91 ^a^	25.67 ± 4.25 ^a^	23.94 ± 2.07 ^a^
8	37.20 ± 0.94 ^a^	40.96 ± 3.61 ^a^	36.94 ± 2.04 ^a^	37.80 ± 3.48 ^a^

All values are mean ± SD. ^a,b^ Values in the same row with different superscript letters are significantly different (*p* < 0.05).

**Table 8:** Water activity of untreated and PDB-treated (3, 5, and 7 kgf/cm^2^) adzuki beans.

Soaking Time, h	Water Activity (Aw)			
	Control	PDB 3 kgf/cm^2^	PDB 5 kgf/cm^2^	PDB 7 kgf/cm^2^
0	0.5671 ± 0.0258 ^c^	0.5335 ± 0.0122 ^ab^	0.5482 ± 0.0012 ^bc^	0.5154 ± 0.0048 ^a^
2	0.6812 ± 0.0773 ^a^	0.7076 ± 0.1036 ^a^	0.7507 ± 0.0859 ^a^	0.7144 ± 0.0264 ^a^
4	0.7945 ± 0.0727 ^a^	0.8414 ± 0.0560 ^a^	0.8490 ± 0.0499 ^a^	0.7933 ± 0.0330 ^a^
6	0.9310 ± 0.0054 ^b^	0.9371 ± 0.0175 ^b^	0.9130 ± 0.0302 ^ab^	0.8762 ± 0.0356 ^a^
8	0.9637 ± 0.0233 ^a^	0.9704 ± 0.0042 ^a^	0.9670 ± 0.0094 ^a^	0.9682 ± 0.0144 ^a^

All values are mean ± SD. ^a,b,c^ Values in the same row with different superscript letters are significantly different (*p* < 0.05).

**Table 9:** Moisture content of untreated and heat-treated (60 and 100 °C) and PDB-treated (25, 60, and 100 °C) adzuki beans.

Soaking Time	Moisture Content (%)					
	Control	Heat 60 °C	Heat 100 °C	PDB 25 °C	PDB 60 °C	PDB 100 °C
0 h	10.21 ± 0.27 ^bc^	10.33 ± 0.26 ^c^	9.75 ± 0.43 ^ab^	10.29 ± 0.13 ^c^	10.18 ± 0.16 ^bc^	9.40 ± 0.26 ^a^
2 h	11.58 ± 0.80 ^a^	11.31 ± 0.08 ^a^	14.19 ± 3.17 ^b^	11.61 ± 0.87 ^a^	13.64 ± 1.89 ^b^	11.24 ± 1.50 ^a^
4 h	15.55 ± 1.56 ^a^	17.25 ± 4.50 ^a^	17.58 ± 3.17 ^a^	16.19 ± 2.26 ^a^	20.22 ± 2.75 ^a^	17.58 ± 3.07 ^a^
6 h	24.12 ± 0.82 ^ab^	26.00 ± 1.64 ^ab^	23.01 ± 3.61 ^a^	25.67 ± 4.25 ^ab^	27.40 ± 3.65 ^ab^	29.21 ± 4.04 ^b^
8 h	37.20 ± 0.94 ^a^	41.84 ± 3.94 ^b^	42.31 ± 1.59 ^b^	36.94 ± 2.04 ^a^	38.95 ± 1.71 ^ab^	41.33 ± 3.43 ^b^

All values are mean ± SD. ^a,b,c^ Values in the same row with different superscript letters are significantly different (*p* < 0.05).

**Table 10:** Water activity of untreated and heat-treated (60 and 100 °C) and PDB-treated (25, 60, and 100 °C) adzuki beans.

Soaking Time	Water Activity (Aw)					
	Control	Heat 60 °C	Heat 100 °C	PDB 25 °C	PDB 60 °C	PDB 100 °C
0 h	0.5671 ± 0.0258 ^bc^	0.5374 ± 0.0040 ^a^	0.5612 ± 0.0015 ^abc^	0.5482 ± 0.0012 ^ab^	0.5385 ± 0.0074 ^a^	0.5792 ± 0.0043 ^c^
2 h	0.6812 ± 0.0773 ^a^	0.6709 ± 0.0865 ^a^	0.7350 ± 0.1382 ^a^	0.7507 ± 0.0859 ^a^	0.7027 ± 0.0586 ^a^	0.7275 ± 0.0730 ^a^
4 h	0.7945 ± 0.0727 ^a^	0.7821 ± 0.0720 ^a^	0.8323 ± 0.0372 ^a^	0.8490 ± 0.0499 ^a^	0.8578 ± 0.0452 ^a^	0.8819 ± 0.0309 ^a^
6 h	0.9310 ± 0.0054 ^ab^	0.9288 ± 0.0213 ^ab^	0.9112 ± 0.0083 ^a^	0.9130 ± 0.0302 ^ab^	0.9548 ± 0.0106 ^ab^	0.9534 ± 0.0270 ^b^
8 h	0.9637 ± 0.0233 ^ab^	0.9502 ± 0.0100 ^a^	0.9637 ± 0.0080 ^ab^	0.9670 ± 0.0094 ^ab^	0.9775 ± 0.0058 ^b^	0.9773 ± 0.0085 ^b^

All values are mean ± SD. ^a,b,c^ Values in the same row with different superscript letters are significantly different (*p* < 0.05).

**Table 11:** Moisture content of untreated and PDB-treated (10, 40, and 100 times) adzuki beans.

Soaking Time, h	Moisture Content (%)			
	Control	PDB 10 Times	PDB 40 Times	PDB 100 Times
0	10.21 ± 0.27 ^a^	10.29 ± 0.13 ^a^	10.22 ± 0.23 ^a^	10.54 ± 0.46 ^a^
2	11.58 ± 0.80 ^a^	11.61 ± 0.87 ^a^	11.17 ± 0.20 ^a^	10.53 ± 0.24 ^a^
4	15.55 ± 1.56 ^a^	16.19 ± 2.26 ^ab^	18.71 ± 0.60 ^b^	16.48 ± 0.98 ^ab^
6	24.12 ± 0.82 ^a^	25.67 ± 4.25 ^a^	28.66 ± 6.07 ^a^	29.47 ± 5.49 ^a^
8	37.20 ± 0.94 ^a^	36.94 ± 2.04 ^a^	37.90 ± 0.77 ^a^	41.76 ± 3.04 ^b^

All values are mean ± SD. ^a,b^ Values in the same row with different superscript letters are significantly different (*p* < 0.05).

**Table 12:** Water activity of untreated and PDB-treated (10, 40, 100 times) adzuki beans.

Soaking Time, h	Water Activity (Aw)			
	Control	PDB 10 Times	PDB 40 Times	PDB 100 Times
0	0.5671 ± 0.0258 ^b^	0.5482 ± 0.0012 ^ab^	0.5355 ± 0.0043 ^a^	0.5436 ± 0.0104 ^ab^
2	0.6812 ± 0.0773 ^a^	0.7507 ± 0.0859 ^a^	0.6988 ± 0.0693 ^a^	0.6908 ± 0.0617 ^a^
4	0.7945 ± 0.0727 ^a^	0.8490 ± 0.0499 ^a^	0.8317 ± 0.0316 ^a^	0.8252 ± 0.0362 ^a^
6	0.9310 ± 0.0054 ^a^	0.9130 ± 0.0302 ^a^	0.8904 ± 0.0187 ^a^	0.9120 ± 0.0191 ^a^
8	0.9637 ± 0.0233 ^a^	0.9670 ± 0.0094 ^a^	0.9716 ± 0.0223 ^a^	0.9753 ± 0.0031 ^a^

All values are mean ± SD. ^a,b,c^ Values in the same row with different superscript letters are significantly different (*p* < 0.05).

## Data Availability

The original contributions of this study are included in this article. Further inquiries can be directed to the corresponding authors.

## References

[1] Seon-Min O., Young-Je J., Areum C., Jieun K., You-Geun O., Mi-Jung K., Suk-Bo S., Induck C. (2021). Seed and water absorption characteristics of red bean cultivars in Korea. Korean J. Food Sci. Technol..

[2] Yousif A.M., Kato J., Deeth H.C. (2007). Effect of storage on the biochemical structure and processing quality of adzuki bean (*Vigna angularis*). Food Rev. Int..

[3] Ueno S., Shigematsu T., Karo M., Hayashi M., Fujii T. (2015). Effects of high hydrostatic pressure on water absorption of adzuki beans. Foods.

[4] Yousif A.M., Batey I.L., Larroque O.R., Curtin B., Bekes F., Deeth H.C. (2003). Effect of storage of adzuki bean (*Vigna angularis*) on starch and protein properties. Lebensm.-Wiss. Technol..

[5] Piergiovanni A.R. (2011). Kinetic of water adsorption in common bean: Considerations on the suitability of Peleg’s model for describing bean hydration. J. Food Process. Preserv..

[6] An Y.E., Ahn S.C., Yang D.C., Park S.J., Kim B.Y., Baik M.Y. (2011). Chemical conversion of ginsenosides in puffed red ginseng. LWT–Food Sci. Technol..

[7] Kim J., Lee H.I., Lim Y.J., Park Y.J., Kim W., Kim D.O., Kim B.Y., Eom S.H., Baik M.Y. (2020). Antioxidant and phytoestrogenic activities of puffed black soybeans (*Glycine max*). LWT Food Sci. Technol..

[8] Paznocht L., Buresova B., Kotikova Z., Martinek P. (2021). Carotenoid content of extruded and puffed products made of colored-grain wheats. Food Chem..

[9] Eikani M.H., Khandan N., Feyzi E. (2019). Enhancing bio-oil extraction using instant controlled pressure drop texturing. Food Bioprod. Process..

[10] Martinez-Meza Y., Perez-Jimenez J., Rocha-Guzman N.E., Rodriguez-Garcia M.E., Alonzo-Macias M., Reynoso-Camacho R. (2021). Modification on the polyphenols and dietary fiber content of grape pomace by instant controlled pressure drop. Food Chem..

[11] Pliego-Cortés H., Boy V., Bourgougnon N. (2024). Instant Controlled Pressure Drop (DIC) as an innovative pre-treatment for extraction of natural compounds from the brown seaweed *Fensholt 1955*. Algal Res..

[12] Ranjbar N., Eikani M.H., Javanmard M., Golmohammad F. (2016). Impact of instant controlled pressure drop on phenolic compounds extraction from pomegranate peel. Innov. Food Sci. Emerg. Technol..

[13] Cattaneo S., Hidalgo A., Masotti F., Stuknyte M., Brandolini A., De Noni I. (2015). Heat damage and in vitro starch digestibility of puffed wheat kernels. Food Chem..

[14] Cheon S.-H., Eun J.-B. (2011). The physical properties of puffed snacks (*ppeongtuigi*) added with sweet potato flours. J. Appl. Biol. Chem..

[15] Kaur R., Kumar A., Kumar V., Kumar S., Saini R.K., Nayi P., Gehlot R. (2023). Recent advancements and applications of explosion puffing. Food Chem..

[16] Ki-Jung L., Su-Yong L., Yong-Ro K., Jang-Woo P., Jaeyong S. (2004). Effect of dry heating on the pasting/retrogradation and textural properties of starch-soy protein mixture. Korean J. Food Sci. Technol..

[17] Kwan Hwa P., Ze Uook K., Jae Doo S., Bong Soo N. (1979). Thermal inactivation of crude papain and papaya peroxidase. Korean J. Food Sci. Technol..

[18] Dong-Duk Y., Tae-Heok K., Joong-Woo L., Youn-Chul C., Myung-Ryoul O. (2004). Cavitation in tunnel spillways. Tunnel Undergr. Space.

[19] Jung-Hyun N., Ji-Yeon C. (2021). Effect of browning inhibitors NaCl and CaCl₂ on the qualities of Jeju Tamna potatoes during hot-air drying. J. Korean Soc. Food Sci. Nutr..

[20] Jong Dae P., Hyang Mi J., Jun Seok K., Hyun Yu L. (2006). Soaking and drying characteristics of grains and legumes. Food Sci. Preserv..

[21] Deshpande S.S., Cheryan M. (1986). Microstructure and water uptake of *Phaseolus* and winged beans. J. Food Sci..

[22] Miano A.C., Pereira J.D., Castanha N., da Matta M., Augusto P.E.D. (2016). Enhancing mung bean hydration using ultrasound technology: Description of mechanisms and impact on germination and main components. Sci. Rep..

[23] Hu L.A., Bi J.F., Jin X., van der Sman R. (2022). Microstructure evolution affecting the rehydration of dried mushrooms during instant controlled pressure drop combined hot air drying (DIC-HA). Innov. Food Sci. Emerg. Technol..

[24] Mounir S., Allaf T., Mujumdar A.S., Allaf K. (2012). Swell drying: Coupling instant controlled pressure drop (DIC) to standard convection drying processes to intensify transfer phenomena and improve quality—An overview. Dry. Technol..

[25] Mounir S., Amami E., Allaf T., Mujumdar A., Allaf K. (2020). Instant controlled pressure drop (DIC) coupled to intermittent microwave/airflow drying to produce shrimp snacks: Process performance and quality attributes. Dry. Technol..

[26] Mounir S., Besombes C., Al-Bitar N., Allaf K. (2011). Study of instant controlled pressure drop (DIC) treatment in manufacturing snack and expanded granule powder of apple and onion. Dry. Technol..

